# Selenium and Mercury in the Brazilian Amazon: Opposing Influences on Age-Related Cataracts

**DOI:** 10.1289/ehp.0901284

**Published:** 2010-08-17

**Authors:** Mélanie Lemire, Myriam Fillion, Benoît Frenette, Annie Mayer, Aline Philibert, Carlos José Sousa Passos, Jean Rémy Davée Guimarães, Fernando Barbosa, Donna Mergler

**Affiliations:** 1 Centre de recherche interdisciplinaire sur la biologie, la santé, la société et l’environnement, Université du Québec à Montréal, Montréal, Canada; 2 École d’Optométrie, Université de Montréal, Montréal, Québec, Canada; 3 Faculdade UnB-Planaltina, Universidade de Brasília, Brasilía, Brazil; 4 Laboratório de Traçadores, Instituto de Biofísica Carlos Chagas Filho, Universidade Federal do Rio de Janeiro, Rio de Janeiro, Brazil; 5 Laboratório de Toxicologia e Essencialidade de Metais, Departamento de Análises Clínicas, Toxicológicas e Bromatológicas, Faculdade de Ciências Farmacêuticas de Ribeirão Preto, Universidade de São Paulo, Ribeirão Preto, Brazil

**Keywords:** age-related cataract, Brazilian Amazon, fish consumption, mercury, selenium

## Abstract

**Background:**

Age-related cataracts (ARCs) are an important cause of blindness in developing countries. Although antioxidants may be part of the body’s defense to prevent ARC, environmental contaminants may contribute to cataractogenesis. In fish-eating populations of the lower Tapajós region, elevated exposure to mercury (Hg) has been reported, and blood levels of selenium (Se) range from normal to very high (> 1,000 μg/L).

**Objectives:**

We examined ARCs in relation to these elements among adults (≥ 40 years of age) from 12 riverside communities.

**Methods:**

Participants (*n* = 211) provided blood samples and underwent an extensive ocular examination. Inductively coupled plasma mass spectrometry was used to assess Hg and Se in blood and plasma.

**Results:**

One-third (*n* = 69; 32.7%) of the participants had ARC. Lower plasma Se (P-Se; < 25th percentile, 110 μg/L) and higher blood Hg (B-Hg; ≥ 25th percentile, 25 μg/L) were associated with a higher prevalence odds ratio (POR) of ARC [adjusted POR (95% confidence interval), 2.69 (1.11–6.56) and 4.45 (1.43–13.83), respectively]. Among participants with high P-Se, we observed a positive but nonsignificant association with high B-Hg exposure, whereas among those with low B-Hg, we observed no association for P-Se. However, compared with the optimum situation (high P-Se, low B-Hg), the POR for those with low P-Se and high B-Hg was 16.4 (3.0–87.9). This finding suggests a synergistic effect.

**Conclusion:**

Our results suggest that persons in this population with elevated Hg, the cataractogenic effects of Hg may be offset by Se. Because of the relatively small sample size and possible confounding by other dietary nutrients, additional studies with sufficient power to assess multiple nutrient and toxic interactions are required to confirm these findings.

Age-related cataract (ARC) is a leading cause of impaired vision among elderly populations, particularly in developing nations where there is little access to surgical procedures (World Health Organization 1991). ARC is generally characterized by a gradual painless loss of vision from damage to and accumulation, aggregation, and precipitation of lens proteins that cause a partial or complete progressive lens opacification. ARC prevalence increases from approximately 5% at 65 years of age to 50% at 75 years of age (Chiu and Taylor 2007).

The eye’s lens is avascular; it depends mostly on passive diffusion, active transport, and intralens protein synthesis. Compared with other tissues, the lens contains high concentrations of locally synthesized glutathione (GSH) that decrease with age (Reddy and Giblin 1984). Reduced GSH, the “active” form of GSH that is involved in the antioxidant defense system, may act as the first line of protection against cataract formation (Fernandez and Afshari 2008).

Observational and clinical trials have evaluated the potential protective effect of nutrients such as vitamins C and E, carotenoids, and selenium (Se) on lens tissues (Chiu and Taylor 2007; Flohé 2005). Since the 1960s, research has suggested that specific Se-containing enzymes, such as glutathione peroxidase (GPx), may be part of the body’s defense to prevent or delay the progression of ARC (Flohé 2005). Animal studies have shown that chronic Se deficiency or GPx depletion can lead to cataract formation, whereas Se excess can induce prooxidant conditions involved in cataractogenesis (Cai et al. 1994; Reddy et al. 2001; Kyselova 2010). However, the role of Se status in the formation of human cataract remains unclear (Flohé 2005; Li et al. 2009).

ARC pathology is believed to result from a combination of risk factors acting over many years, such as smoking; ultraviolet light; exposure to heavy metals, including cadmium and mercury (Hg); and the use of steroids and gout medication (see Head 2001). For many of these factors, oxidative damage or unbalance in reduced GSH concentrations may be the underlying process leading to degenerative opacities of the lens (Head 2001; Truscott 2005).

At low latitudes, where solar ultraviolet exposure is high, there is an elevated prevalence of ARC (Sasaki et al. 2003). The Tapajós River, a major tributary of the Amazon, is situated near the equator, and inhabitants spend most of the day outdoors, involved in subsistence activities such as traditional fishing, slash-and-burn agriculture, and washing clothes and dishes in the river. The fish-eating populations from this region have among the highest reported Hg exposure in the world today (Passos and Mergler 2008). On the other hand, Se status in these same communities ranges from normal to very high (Lemire et al. 2006, 2009).

The objective of the present study was to examine the prevalence of ARC in relation to Hg exposure and Se status.

## Material and Methods

### Study population

Since the mid-1990s, our research group has been involved in an interdisciplinary project on Hg exposure and its effects on human health in the lower Tapajós River basin (State of Pará, Brazil) (Caruso Project 2010). In this region, there are approximately fifty communities of diverse sizes and origins, with varying access to health care, education, and consumer goods. The results presented here are part of a cross-sectional study that examined factors that may influence Hg toxicity. For this study, we selected twelve communities to reflect the diversity of regional populations, social conditions, and ecosystems. Sample-size calculation, based on visual outcome measurements (near visual contrast sensitivity) from preliminary studies, indicated that we would require a minimum of 400 people to test Hg and Se interaction effects.

Because it is difficult to apply a random sampling strategy in Amazonian riverside communities (Passos et al. 2007), we used a convenience sampling procedure (Barr et al. 2006). Several weeks before the present study, each village was visited by two members of the research team who explained the study at village meetings and home visits. Persons ≥ 15 years of age were invited to participate on a voluntary basis. Participation was limited to a maximum of 12 persons per day because the examination procedure required bringing participants by boat to a technical school in a nearby city where we had access to electricity and freezers for storing biological material. Each village was scheduled for a specific number of days. The boats arrived the previous evening and made the trip during the night. The study was carried out from May to July 2006.

The study was approved by the ethics review boards of the University of Quebec at Montreal and the Faculty of Pharmaceutical Sciences of the University of São Paulo–Ribeirão Preto. All participants signed an informed consent form, which was read to them. No remuneration was provided for study participation.

### Ocular health assessment

Ocular health examinations were performed by four clinical optometrists from the School of Optometry of the University of Montreal. The anterior segment of the eye was examined using slit-lamp biomicroscopy, with normalized slit section and maximal, constant luminal intensity (model SL-J, serial no. 64459, NEITZ Instruments Co., LTD, Tokyo, Japan). The posterior segment of the retina and its periphery were examined during pupillary dilation using two mydriatic agents [tropicamide 1% and phenylephrine 2.5% (ALCON, Mississauga, Ontario, Canada)]. In addition, iridocorneal angles were noted. Intraocular pressure was measured with a hand-held applanation tonometer (Perkins MKII, serial no. 3611764; Clement Clarke Inc., Columbus, OH, USA) under topical anesthesia.

All lens opacities were noted, and ARC was distinguished from other lens pathologies, such as cataracts typical of trauma, which were noted for post hoc exclusions in the present analysis. A severity score, based on opalescence, color, and lens opacity, derived from the Lens Opacities Classification System III (Chylack et al. 1993), was applied. The score ranged from grade 1 (lens with few opacities and a light yellowish nucleus) to grade 4 (brownish nucleus with more opacities).

The four optometrists were trained before the field study to minimize interobserver bias and were blinded to the level of Hg exposure and Se status of the participants. A team of two optometrists performed examinations from 5 May to 6 June, and a second team of two from 4 to 28 July. All examinations were made in the same room and under the same conditions. If there was any doubt regarding a diagnosis, the teams consulted each other and arrived at a consensus. Statistical comparison of cataract cases diagnosed by the two teams showed a similar prevalence [*n* = 33/110 (33.0%) vs. *n* = 36/101 (35.6%); Fisher’s exact test, *p* = 0.46].

For the present analyses, each person was classified with respect to the presence or absence of ARC in at least one eye. To categorize opacity, we attributed the most severe grade when there was a difference in the two eyes.

### Blood sample collection and analysis

For each participant, an experienced Brazilian phlebotomist collected a 6-mL blood sample in trace-metal free, evacuated tubes (BD-Brasil, São Paulo, Brazil), containing heparin as an anticoagulant. For plasma separation, blood samples were centrifuged (Excelsa II model 206 BL; FANEM, Guarulhos, São Paulo, Brazil) (800 × *g* for 6 min). Plasma fractions were then pipetted into previously cleaned Eppendorf tubes (2 mL) and immediately frozen at −20°C. Blood total Hg (B-Hg), blood Se (B-Se), plasma total Hg (P-Hg), and plasma Se (P-Se) were determined by inductively coupled plasma mass spectrometry (ICP-MS; DRC II, Perkin Elmer Sciex, Shelton, CT, USA) according to the method proposed by Batista et al. (2009), at the Laboratório de Toxicologia e Essencialidade de Metais, Universidade de São Paulo, (Ribeirão Preto, São Paulo, Brazil). Quality control was guaranteed by analyzing various secondary reference materials, such as BE 06-01, BE 06-02, BE 06-03, BE 06-05, QMEQAS 06B06, and QMEQAS 06B03, that were provided by the New York State Department of Health’s proficiency testing program for trace elements in whole blood (New York State Department of Health 2006), and from the external quality assessment scheme for trace elements operated by the Institut National de Santé Publique du Québec, Canada (Institut national de santé publique, Quebec 2007). Reference materials were analyzed before and after 10 ordinary samples. Measured values were always within the provided reference or certified values.

### Questionnaires and medication

Sociodemographic characteristics, including age, sex, current smoking habits, alcohol consumption, and medical history, were surveyed using an interview-administered questionnaire that was adapted from our previous studies (Lebel et al. 1998; Dolbec et al. 2000). Research assistants visited all the participants in their homes where they recorded the name of all the medications that they were taking at the time of the interview.

### Statistical analyses

Descriptive statistics were used to illustrate the study population’s general characteristics, the distribution of the biomarkers, the prevalence of ARC and the distribution of ARC grading. Because none of the biomarker variables displayed a normal distribution, we performed nonparametric analyses. The rank sums test (Wilcoxon and Kruskall–Wallis chi-squared tests) was performed to compare continuous variables between groups, and Spearman’s ρ was used to evaluate the correlations between two continuous variables. We retained two-tailed Fisher’s exact test for the analyses of contingency tables.

Multivariate logistic regression models and prevalence odds ratios (PORs) with 95% confidence intervals (CIs) were used to examine, separately and jointly, the associations between ARC and covariables, such as age (as a continuous variable), sex, current smoking habit (yes vs. no), and alcohol consumption (yes vs. no). Examiner team (2 vs. 1) was always included in the models. Because no *a priori* information was available to indicate where a cutoff effect, if present, would occur, we proceeded in several steps. First, despite the small sample size, we categorized biomarkers by quartiles and used logistic regression models (crude and adjusted) to examine, for each biomarker, the relation with ARC, taking into account the relevant covariables. These results were subsequently used to regroup quartiles into binary categories that allowed us to estimate associations with the biomarkers (as binary variables) in the same model. Because possible modifying effects of Se on Hg toxicity have been suggested (Khan and Wang 2009), we examined different Se and Hg combinations to evaluate possible additive, multiplicative, or synergistic effects. Results were defined as statistically significant at *p* < 0.05. Analyses were performed using JMP 8.0.1 (SAS Institute Inc., Cary, NC, USA ) and SPSS 16.0 (SPSS Inc., Chicago, IL, USA).

## Results

A total of 448 persons (216 men and 232 women) agreed to participate. Reasons for nonparticipation included work, domestic responsibilities, religious convictions, illnesses, and lack of interest in the study. We compared the age distribution of the participants with that of the total population based on a house-to-house survey that was carried out in 2003. The participants in the present study represent 25% of the adult population of these villages (27% of all women and 23% of all men). The proportion of persons from each village varied from 10–67%, with higher relative frequencies from the smaller villages (the smallest consisted of 13 adult residents and the largest, 384 adults). Younger persons (≥ 15 and < 40 years) were underrepresented with respect to the age distribution of the entire population (50% vs. 62%), those 40–65 years of age were overrepresented (40% vs. 28%), whereas representation of the oldest group (≥ 65 years) was similar to the total population (10%).

Because only two persons < 40 years of age had cataracts, we restricted the present study to participants ≥ 40 years of age (*n* = 221). We applied as post hoc exclusions persons who reported a diagnosis of diabetes (*n* = 4) and those who were taking steroid medication (*n* = 4). No participants reported gout or taking gout-related medication. We also excluded two participants who had had intraocular lens (cataract) surgery in both eyes. We did not exclude one participant who had had cataract surgery on the right eye and had grade 4 ARC in the left eye. We included a total of 211 persons, 96 women and 115 men, in the present analyses.

The ages of the participants ranged from 40–87 years (mean ± SD = 55.1 ± 10.8 years; median = 54 years). Consistent with our knowledge of ARC, we categorized participants into two age groups: 40–65 years (*n* = 171) and ≥ 65 years (*n* = 40; Table 1). The distribution of men and women was similar between and within age groups; age distribution was similar for men and women. Forty-four percent of the study population reported drinking alcohol; there were fewer alcohol drinkers in the older group than in the younger group (27.5% vs. 47.3%; *p* = 0.03). Among drinkers, the majority (72.8%) indicated drinking only on special occasions, whereas for the others, median consumption was three alcoholic beverages/week. Thirty percent of the study population reported currently smoking, and the proportion of smokers was comparable between age categories. Among smokers, the median frequency of smoking was low (five cigarettes/day).

ARC in at least one eye was diagnosed in 69 persons (32.7%). As expected, ARC prevalence was significantly higher among persons in the older age group (82.5% vs. 21.1%, *p* < 0.0001; Table 1). We found no significant difference for ARC prevalence among men and women overall or within the age groups. ARC was more severe among older persons (Table 1).

Median levels (range) for the biomarkers were B-Se, 222 μg/L (124–1,500 μg/L); P-Se, 133 μg/L (57–913 μg/L); B-Hg, 44 μg/L (4.3–289 μg/L); P-Hg, 6.4 μg/L (0.2–40.0 μg/L). Biomarkers of Se decreased with age, but Hg biomarkers were not correlated with age (Table 2). B-Se and P-Se were positively correlated with B-Hg but not with P-Hg. B-Hg concentrations were higher among men than among women (median = 44.0 vs. 43.0 μg/L), but the difference was not statistically significant (*p* = 0.07). Biomarkers of Se were significantly higher among those who reported drinking alcohol than among those who did not (B-Se, 241 vs. 212 μg/L; P-Se, 126 vs. 91 μg/L; *p* < 0.01), whereas biomarkers of Hg were higher among smokers than among nonsmokers (B-Hg, 46 vs. 43 μg/L; P-Hg, 7.2 vs. 6.2 μg/L; *p* < 0.05).

We estimated PORs and 95% CIs for ARC in association with relevant covariables (age as a continuous variable, sex, smoking, drinking, and examiner team) using bivariate and multivariate logistic regression models. In the bivariate models, ARC was positively associated with age (POR = 1.20; 95% CI, 1.14–1.27) and negatively with alcohol consumption (POR = 0.48; 95% CI, 0.26–0.88). We observed no associations between ARC and the other covariables. In a multivariate model that included all the covariables above, only age was significantly associated with ARC (POR = 1.23; 95% CI, 1.16–1.30).

P-Se was more strongly associated with ARC than was B-Se, with a significant inverse adjusted POR for the second versus first quartile and inverse but not significant PORs for the third and fourth quartiles relative to the first (Table 3). Adjusted PORs were not significant for B-Se. B-Hg was positively associated with ARC at the second and third versus first quartiles; although the POR for fourth versus first B-Hg quartile was elevated, it was not statistically significant and was lower than the other effect estimates. We observed no associations between P-Hg and ARC.

For the analyses presented in Table 4, we grouped the upper three quartiles of P-Se (high P-Se) and compared them with the first quartile (low P-Se), and we grouped the upper three quartiles of B-Hg (high B-Hg) and compared them with the first quartile (low B-Hg). The adjusted ARC prevalence was significantly higher for those with low versus high P-Se and for those with high versus low B-Hg. Comparing different combinations of high and low P-Se and B-Hg with the optimum condition high P-Se–low B-Hg showed that for the combination low P-Se–low B-Hg, there was no difference in ARC prevalence, whereas for the combination high P-Se–high B-Hg, we found a positive but nonsignificant difference in ARC. Finally, when comparing persons with low P-Se–high B-Hg with persons with the optimum conditions (high P-Se–low B-Hg), the POR is very high. This result suggests a synergistic effect rather than an additive or multiplicative effect.

It is noteworthy that the adjusted POR (95% CI) is 13.5 (2.23–81.6) when comparing persons with low P-Se–high B-Hg and persons with low P-Se–low B-Hg, and 5.09 (1.67–15.1) when comparing persons with low P-Se–high B-Hg and persons with high P-Se–high B-Hg, revealing a significant association between ARC and B-Hg when P-Se is low, and a significant association between ARC and P-Se when B-Hg is high.

Because of the high prevalence of ARC among the older participants (82.%), we examined ARC prevalence separately in 40- to 65-year-olds (all ARC grades combined vs. no ARC) and persons ≥ 65 years of age (grade 4 ARC vs. all others). We used the median value of B-Hg (44 μg/L) to ensure a better distribution in each quadrant. ARC was most prevalent among persons with high B-Hg and low P-Se in both age groups. The adjusted POR patterns for both age groups were similar to the overall group, although estimates were imprecise, and few were statistically significant for the older group.

## Discussion

For this population in the Brazilian Amazon, where environmental exposure to Hg is elevated compared with most areas in the world (Passos and Mergler 2008), the prevalence of ARC varied in opposite directions in association with P-Se and B-Hg. We observed the lowest prevalence for those with high P-Se and low B-Hg, and the highest prevalence for those with high B-Hg and low P-Se levels. The high POR between the “worst” and the “optimum” exposure combinations suggests possible antagonism for the protective effects of Se and the adverse effects of Hg. It is noteworthy that for persons with high P-Se, we observed a moderate nonsignificant association between B-Hg and ARC, whereas for those with low B-Hg, no association was found between P-Se and ARC.

The positive association between ARC and B-Hg for those with lower P-Se is a new finding. Few studies have examined cataracts in relation to Hg exposure, although some scientists have suggested that Hg accumulates in the lens and may be involved in cataract formation (Gabal and Raslan 1995; Winder et al. 1980). Reddy and Giblin (1984) showed that pretreatment of human and rabbit lenses with methylmercury decreased reduced GSH concentration by 75%; the lenses were also less effective in hydrogen peroxide conversion, which can result in lens opacification. Indeed, Hg molecules have a high affinity to sulfhydryl groups of small molecules, such as reduced GSH and cysteine proteins (see Clarkson and Magos 2006). Some researchers have suggested that Hg can also bind to Se in the active site of selenoenzymes, such as GPx, thereby inhibiting their enzymatic functions (Seppänen et al. 2004). For indigenous populations in the Amazon, Paula et al. (2006) reported a higher prevalence of ARC for the groups that depend on fishing than for those that depend on hunting (24.5% vs. 13.7%), but no information is provided on Hg exposure.

The literature on the association between Se and ARC in human populations is inconsistent. For example, Swanson and Truesdale (1971) reported low levels of Se in cataractous human lens tissues. Karaküçük et al. (1995) reported lower Se concentrations in the aqueous humor of patients with ARC, but they found no differences in Se lens content. Some epidemiological studies have indicated that Se status is negatively associated with ARC (Karaküçük et al. 1995; Valero et al. 2002), whereas one study observed a positive association with B-Se (Jacques et al. 1988). Akesson et al. (1987) observed no association with P-Se levels among 68-year-old men with and without cataracts, but mean P-Se was considerably lower than in our study (~ 85 μg/L). Li et al. (2009) reported no difference in ARC prevalence between persons living in a poor- and a rich-Se area. Several factors may explain differences among studies, including variation in the range of Se status, the use of different biomarkers of Se status, differences in the chemical form of the Se intake (inorganic vs. organic), and the influence of confounding factors and concomitant environmental and occupational exposures.

In the present study, it is not clear whether the Se association with ARC was independent of Hg-related lens damage. ARC was strongly associated with B-Hg exposure among those with P-Se status < 110 μg/L, but the prevalence of ARC did not significantly increase with Hg exposure among those whose P-Se was equal to or above this concentration. Several selenoproteins may be involved in lens protection against reactive oxygen species that cause protein cross-linking and lipid peroxidation in the lens (see Flohé 2005). Cytosolic GPx (GPx-1) uses GSH as reducing substrate and has been shown to play a central role in hydrogen peroxide detoxification and, consequently, against cataract formation. Together with glutathione reductase and glutathione synthase, GPx-1 is involved in reduced GSH pool regeneration in the lens. Extracellular GPx (GPx-3), which plays a role in the regulation of extracellular hydrogen peroxide, has also been identified in the eye (Flohé 2005). Recent evidence suggests that the Se intake required to optimize all different selenoproteins would require P-Se concentrations around 125 μg/L (Burk et al. 2006).

Two hypotheses may explain our findings. First, Se may play an important preventive role for ARC disease through its antioxidant enzymatic activities; second, higher Se intake may be essential to counter the effects of Hg-induced cataracts by restoring the selenoenzymes or the GSH pool. A combination of both is also possible. Because in the present study no one is exempt from Hg exposure, we are unable to conclude whether higher Se status (in the absence of Hg) can prevent or delay the progression of ARC.

As expected, the prevalence of cataracts increased with age. It is noteworthy that those with low Se and high Hg in the 40- to 65-year age category displayed an ARC prevalence similar to that for ≥ 65-year-olds, although the severity of ARC was greater among persons in the older group. For those ≥ 65 years of age, the POR in the group with the highest risk (low P-Se–high B-Hg) was elevated but did not reach significance, at least in part due to the small numbers of observations in this age category.

Cataracts have been induced in experimental models with high concentrations of inorganic Se (selenite), at doses below those causing acute Se systemic toxicity (Flohé 2005). In the present study, we observed no adverse effects although Se concentrations were very high, reaching 1,500 μg/L for B-Se and 913 μg/L for P-Se. In this riverside population, high Se intake comes from local diet, which contains mostly organic forms of Se (Lemire et al. 2010). The toxic effects of organic Se are less understood, and toxicity, if it exists, may occur at higher levels compared with inorganic Se (Rayman et al. 2008). On the other hand, concomitant exposure to metals, such as Hg, may raise the body’s Se requirements to offset toxic effects (Fordyce 2005; Watanabe 1999) and to maintain optimal Se antioxidant enzymes and other Se physiological activities (Rayman 2009). Thus, in a situation with elevated Hg and Se, there may be less “excess” of Se and consequently little or no Se toxicity.

B-Hg was more strongly associated with ARC than was P-Hg. Hg accumulates in the erythrocytes (Clarkson and Magos 2006), and the largest fraction of Hg is measured in whole blood. On the other hand, P-Se was the Se biomarker that was most strongly associated ARC prevalence. Although P-Se reflects recent intake (Combs 2001), Se remains high throughout the year in this population, despite some seasonal variation (Lemire et al. 2009). More than 40% of the Se in human plasma is bound to selenoprotein P (SelP) (Moschos 2000). Plasma SelP is the main transport form of Se for delivery and supporting essential physiological functions of Se in kidney, testis, and brain. However, its role in Se transport to the lens and expression within the lens remains to be established. The lens is an isolated system where most of the proteins, such as selenoproteins and GSH, are probably locally synthesized (Head 2001).

This cross-sectional study suggests that there are opposing effects of P-Se and B-Hg on ARC; however, longitudinal and case–control studies would be useful to confirm these findings and support causal associations. In the Amazon, intense sunlight is omnipresent in daily activities, and because there is little interindividual variability, it would be impossible to control for this important etiological factor in ARC disease (Head 2001).

One of the limits of the present study is that other dietary nutrients may confound the observed associations. Habitants of the lower Tapajós region regularly eat a diversity of fruits, and, when available, vegetables complete the daily food intake (Passos et al. 2001). Fruits and green leafy vegetables can contain high concentrations of beneficial antioxidants (Se, vitamins C, E, beta-carotene), as well as other phytonutrients. Increasing evidence suggests that high consumption of vitamins C and E, and lutein and zeaxanthin carotenoids may help prevent or delay age-related eye diseases, including cataracts (Chiu and Taylor 2007; Fernandez and Afshari 2008). In this population, Brazil nuts are the main source of Se (Lemire et al. 2010). These nuts can also contain high levels of vitamin E and unsaturated fatty acids (Ryan et al. 2006). Therefore, vitamin E may be a confounding factor in the present study. Future studies need to consider a wide range of beneficial nutrients and their potential interactions with toxic elements.

The present study was not based on a random sample, and the ARC prevalence does not necessarily reflect that of the entire population of this region. Using a random sampling strategy in four indigenous communities of the Brazilian Amazon, Paula et al. (2006) reported a prevalence rate of 50.2% for the elderly, but they were unable to determine actual age. In a population-based study of persons ≥ 80 years of age living in Rio Grande do Sul, the southernmost state of Brazil, Romani (2005) reported that 85.6% suffered from cataracts. This proportion is similar to that of participants aged 65–80 years in the present study population (81.5%), suggesting that the conditions in the Amazonian region may increase the risk of ARC. In the United States, the prevalence of ARC during each decade between 40 and 80 years of age is estimated at 2.5%, 6.8%, 20%, and 42.8%, respectively (Eye Diseases Prevalence Research Group 2004).

In the Amazon, riverside populations have little access to surgical cataract repair, and severe ARC is an important cause of blindness among older persons. Public health interventions to alleviate this disease need to consider the risks and benefits of consumption of fish (the major source of Hg) and local foods that are high in Se.

## Figures and Tables

**Table 1:** Characteristics of the study population.

Characteristic	≥ 40 to < 65 years (*n* = 171)	≥ 65 years (*n* = 40)
Women, *n* (%)	80 (46.8)	16 (40.0)
Men, *n* (%)	91 (53.2)	24 (60.0)
Age, years, median (range)	50 (40–64)	73 (65–87)
Women	49 (40–62)	73 (65–84)
Men	52 (40–64)	71 (65–87)
Alcohol drinkers, *n* (%)	81 (47.3)	11 (27.5)
Current smokers, *n* (%)	53 (31.0)	11 (27.5)
ARC,^a^*n* (%)	36 (21.1)	33 (82.5)
Women	14 (17.5)	15 (93.8)
Men	22 (24.2)	18 (75.0)
ARC gradation among cases, *n* (%)		
Grade 1	11 (30.6)	1 (3.0)
Grade 2	18 (50.0)	4 (12.1)
Grade 3	3 (8.3)	6 (18.2)
Grade 4	4 (11.1)	22 (66.7)

a ARC prevalence considering all different grades of ARC.

**Table 2:** Spearman’s ρ correlations (*p*-values) between age and biomarkers and between biomarkers.

	Biomarker			
Measure	B-Se	P-Se	B-Hg	P-Hg
Age, years	−0.30 (< 0.0001)	−0.29 (< 0.0001)	0.03 (0.68)	−0.06 (0.41)
Biomarker				
B-Se		0.72 (< 0.0001)	0.16 (0.02)	−0.02 (0.82)
P-Se			0.18 (0.01)	0.09 (0.19)
B-Hg				0.70 (< 0.0001)

**Table 3:** ARC prevalence and crude and adjusted POR (95% CI) for ARC with respect to biomarker quartiles.

	Biomarker quartile			
Biomarker	First	Second	Third	Fourth
B-Se (μg/L)	124 to < 183	185 to < 222	222 to < 294	287 to 1,500
ARC cases/noncases (*n*)	25/26	20/34	11/42	13/40
Crude POR	1.00	0.61 (0.28–1.33)	0.27 (0.12–0.65)	0.34 (0.15–0.78)
Adjusted^a^ POR	1.00	0.81 (0.27–2.41)	0.64 (0.20–2.04)	1.06 (0.34–3.29)
P-Se (μg/L)	57 to < 110	110 to < 133	133 to < 168	168 to 913
ARC cases/noncases (*n*)	29/23	13/40	16/37	11/42
Crude POR	1.00	0.26 (0.11–0.60)	0.34 (0.15–0.77)	0.21 (0.09–0.50)
Adjusted^a^ POR	1.00	0.20 (0.06–0.64)	0.63 (0.21–1.90)	0.42 (0.13–1.31)
B-Hg (μg/L)	4.3 to < 25	25 to < 44	44 to < 71	71 to 289
ARC cases/noncases (*n*)	12/40	17/36	21/32	19/34
Crude POR	1.00	1.54 (0.66–3.74)	2.19 (0.94–5.11)	1.86 (0.79–4.38)
Adjusted^a^ POR	1.00	4.10 (1.07–15.5)	6.56 (1.78–24.2)	3.29 (0.89–12.1)
P-Hg (μg/L)	0.2 to < 3.3	3.3 to < 6.4	6.4 to < 11	11 to 40
ARC cases/noncases (*n*)	17/34	19/33	11/43	22/32
Crude POR	1.00	1.15 (0.51–2.59)	0.51 (0.21–1.24)	1.38 (0.62–3.05)
Adjusted^a^ POR	1.00	1.60 (0.85–5.13)	0.97 (0.30–3.18)	1.74 (0.56–5.40)

a Adjusted for age, sex, current smoking, alcohol consumption, and examiner team.

**Table 4:** Adjusted PORs with respect to P-Se and B-Hg groups.

Biomarker	ARC cases^a^	Noncases^b^	Adjusted POR (CI 95%)^c^
Age ≥ 40 years			
P-Se			
High (≥ 110 μg/L)	40	119	1.0
Low (< 110 μg/L)	29	23	2.69 (1.11–6.56)

B-Hg			
Low (< 25 μg/L)	12	40	1.0
High (≥ 25 μg/L	57	102	4.45 (1.43–13.83)

Combined P-Se and B-Hg			
High P-Se–low B-Hg	6	28	1.0
Low P-Se–low B-Hg	6	12	1.21 (0.17–8.51)
High P-Se–high B-Hg	34	91	3.22 (0.76–13.6)
Low P-Se–high B-Hg	23	11	16.4 (3.04–87.9)

Age ≥ 40 to < 65 years			
P-Se			
High (≥ 110 μg/L)	21	115	1.0
Low (< 110 μg/L)	15	20	3.53 (1.31–9.45)

B-Hg			
Low (< 44 μg/L)	11	71	1.0
High (≥ 44 μg/L)	25	64	2.17 (0.88–5.55)

Combined P-Se and B-Hg			
High P-Se–low B-Hg	7	57	1.0
Low P-Se–low B-Hg	4	14	1.82 (0.38–8.82)
High P-Se–high B-Hg	14	58	1.65 (0.54–5.07)
Low P-Se–high B-Hg	11	6	11.8 (2.52–55.5)

Age ≥ 65 years			
P-Se			
High (≥ 110 μg/L)	12	11	1.0
Low (< 110 μg/L)	10	7	2.34 (0.42–13.2)

B-Hg			
Low (< 44 μg/L)	12	11	1.0
High (≥ 44 μg/L)	10	7	2.27 (0.36–14.1)

Combined P-Se and B-Hg			
High P-Se–low B-Hg	6	6	1.0
Low P-Se–low B-Hg	6	5	1.21 (0.12–12.1)
High P-Se–high B-Hg	6	5	1.32 (0.14–12.9)
Low P-Se–high B-Hg	4	2	10.1 (0.49–208.4)

a Age ≥ 40 years and ≥ 40 to < 65 years, any ARC; age ≥ 65 years, grade 4 ARC only.

b Age ≥ 40 years and age ≥ 40 to < 65 years, no ARC; age ≥ 65 years, no ARC or ARC < grade 4.

c Adjusted for age, sex, current smoking, alcohol consumption, and examiner team.
